# Finding new and better treatments for psychiatric disorders

**DOI:** 10.1038/s41386-023-01690-5

**Published:** 2023-08-15

**Authors:** Steven M. Paul, William Z. Potter

**Affiliations:** 1 Karuna Therapeutics, Washington University School of Medicine, St. Louis, MO USA; 2 Independent Consultant, Philadelphia, PA USA

**Keywords:** Neuroscience, Psychology

## Abstract

In contrast to most fields of medicine, progress to discover and develop new and improved psychiatric drugs has been slow and disappointing. The vast majority of currently prescribed drugs to treat schizophrenia, mood and anxiety disorders are arguably no more effective than the first generation of psychiatric drugs introduced well over 50 years ago. With only a few exceptions current psychiatric drugs work via the same fundamental mechanisms of action as first-generation agents. Here we describe the reasons for this slow progress and outline a number of areas of research that involve a greater reliance on experimental therapeutics utilizing recent advances in neuroscience to better understand disease biology. We exemplify the potential impact of these areas of research focus with several recent examples of novel agents that have emerged and which support our optimism that newer, more effective and better tolerated agents, are on the horizon. Together with existing drugs these newer agents and novel mechanisms could offer markedly improved functional outcomes for the millions of people still disabled by psychiatric disorders.

## Historical perspective

It remains the case that the majority of pharmacologic treatments for the major psychiatric disorders were initially discovered through serendipity and astute empiricism on the part of clinicians. Following the introduction of first-generation antipsychotics and antidepressants developed this way in the 1950s and 1960s, the significant advances in molecular pharmacology in the 1970s and 1980s provided the “mechanistic” foundation for subsequent identification of the many psychiatric drugs on the market today, that nonetheless work via one or more of the same molecular mechanisms of action (MOA) of the first-generation compounds. The major advances since the introduction of these first-generation agents for both schizophrenia and mood disorders have been primarily in improved tolerability and safety but not in enhanced efficacy. Side effect profiles have benefited from developing compounds that retained elements needed for efficacy while eliminating pharmacologic activities that produce some of the more common side effects. Unfortunately, despite decades of work, the current drugs for treating psychiatric disorders, and their overall efficacy, are arguably no better, especially at improving functional outcomes, than the very first agents introduced well over 50 years ago!

As we will discuss, this disappointing and rather sobering reality has recently begun to change for the better with the emergence of several novel (mechanistically speaking) potential new drugs and approaches for treating schizophrenia and various mood disorders. Here we discuss some of the historical challenges and new opportunities for discovering novel and improved psychiatric drugs.

## The problem

The problem simply stated is that there is too little known about the etiology and pathophysiology of the major psychiatric disorders. Our current, rather limited knowledge of disease biology has not yet meaningfully informed our attempts to find better therapeutics. It was optimistically believed that the great progress in molecular pharmacology (i.e. the application of molecular biology to pharmacology) resulting in the identification of a large number of neurotransmitter receptor families, ion channels, and enzymes as potential drug targets, combined with the modern tools of industrialized drug development, e.g. synthesis of large chemical libraries amenable to high throughput screening, combined with subsequent optimization of leads through modern medicinal chemistry, followed by toxicology and metabolic studies and an understanding of basic pharmacokinetics, would lead to a range of new and more effective medications for those who did not respond well to the original antipsychotics, antidepressants or anxiolytics. There was also the early success story in Parkinson’s disease where the well characterized loss of dopamine neurons and dopamine in the substantia nigra and basal ganglia gleaned from postmortem studies led directly to the use of L-dopa and other dopamine receptor agonists to effectively control the motor symptoms of the disease [1]. Almost two decades of research starting in the early 1970’s focused on the possibility that similar biochemically-defined subgroups of patients with various psychiatric disorders could be identified by measuring monoamine and other neurotransmitters and(or) their metabolites in brain, CSF, blood, or urine. Despite many positive reports from rather small studies supporting this possibility, none of these findings proved sufficiently robust or reproducible to inform the development of new treatments.

Another source of initial optimism was the revolutionary progress being made in genetics and genomics, including the successful sequencing of the human genome and the identification and cataloguing of a very large number of coding and non-coding DNA sequence variants for conducting genome-wide association studies (GWAS) and other genetic studies. Many of us believed that either causative genes or major risk alleles for conditions with high heritability such as schizophrenia and bipolar disorder would be rapidly identified and allow for a specific pathophysiological process to be targeted. Potential drug targets emerging from these genomic approaches could, in theory, follow a linear path from identification to validation and testing with the drug discovery tools that have become available to both academia and industry over the last decade. Unfortunately, such a linear path is not possible with highly polygenic disorders where many individual genes have been shown to contribute very small effects to the disease phenotype or to the risk of developing a DSM-diagnosed syndromal disorder, in contrast to disorders where only one or at most a few causal genes lead to readily identified biochemical and physiological abnormalities that can be corrected pharmacologically. And even if/when there were a demonstrated genetic etiology to a psychiatric disorder such as schizophrenia or bipolar disorder, a linear path to developing effective treatments may prove elusive [2]. For example, while the genetics of schizophrenia are unquestionably complex and highly polygenic [3] there are recent reports of ultra-rare mutations that confer substantial risk of developing schizophrenia and several of these represent “loss of function“ mutations in genes encoding specific glutamate receptor subunits for both N-methyl-D-aspartate (NMDA) and α amino-3-hydroxy-5-methyl-4-isoxazolepropionic acid (AMPA) receptors that are of plausible etiologic relevance to schizophrenia and also highly druggable [4]. However, exactly how these genes contribute to the risk of developing schizophrenia and how drugs acting at these receptors might be used to effectively “treat“ the disease is far from certain and maybe less straightforward than anticipated. For example, if schizophrenia is a neurodevelopmental disorder as has been postulated [5], would these drugs need to be administered at a critical stage of neurodevelopment and would such drugs still be effective years later when symptoms have emerged? It’s also unclear which of the core symptoms of schizophrenia these genetic mutations actually contribute to. To date, drugs designed to enhance NMDAR function have proved of little value in treating any of the core symptoms of schizophrenia. Would they have been more effective if used earlier in its pathogenesis?  Unfortunately, the hope that genetic discoveries would rapidly yield compelling targets for drugs with novel MOAs to correct underlying etiology/pathophysiology has in psychiatry been largely unfulfilled. Other powerful tools have also emerged but have yet to prove instrumental in finding new drugs. These include neuroimaging methods to quantify target engagement by drugs in living subjects for example using positron emission tomography (PET) tracers i.e. to quantify receptor occupancy or using electroencephalography (pharmaco EEG) and functional magnetic resonance imaging (fMRI) to measure the effects of drugs on brain activity and neural circuits associated with a given psychiatric disorder (see below) [6].

Another relevant drug discovery discipline of significant scientific and intellectual pursuit over now several decades has been in what we describe as “behavioral pharmacology“, an extension of behavioral neuroscience, where drugs are systematically studied to impact various observed and readily quantifiable behaviors in nonhuman species, mostly (but not limited to) rodents (i.e. rats and mice). Although these studies have provided very useful information and are almost always employed in any drug discovery effort in psychiatry, their practical value for the discovery of novel psychiatric drugs has, with just a few exceptions, been limited.

Moreover, these behavioral studies have also resulted in what many have mistakenly described as “animal models” of a given disorder, purportedly validated by drugs that have been shown to be effective in various psychiatric disorders and thus where novel mechanisms and agents can then be studied, with the hope that the behavioral findings with these new drugs will ultimately prove translatable to human psychiatric disorders. Such animal models usually involve a behavioral “stressor” or administration of a drug which mimics, in some respect, a particular psychiatric symptom. These models have been developed for most of the psychiatric syndromes, including anxiety, mood, and psychotic disorders [7]. While certain aspects of complex psychiatric syndromes may be “modeled” in such a manner in animals, there are clear limitations. The obvious anthropomorphic limitation of these models of course is that they require a “leap of faith“ as to whether an observed behavior in a rodent can truly reflect any complex emotional disorder in a human being, and especially those characterized by emotions/feelings and behaviors that cannot be readily assessed in non-human species. More recently, genetically modified mice have been used to both identify and confirm the exact mechanisms of drug action and to incorporate genetic mutations believed or shown to contribute to disease etiology/pathophysiology. For example, by genetically eliminating the gene encoding a specific receptor target one can be fairly certain that the behavioral consequences of a given drug, if also eliminated, were in fact due to that receptor. While theoretically powerful, especially when coupled with the advances in molecular pharmacology described above, there have been to date only a few examples where novel psychiatric drugs have been discovered in this way (see below). Moreover since these animal  models have been mostly “validated“ with drugs that predominately work via the same MOA they are really mostly suited for discovering “me too“ iterations of existing agents.

By contrast, genetically modified mice expressing disease-causing mutations or risk alleles have been used extensively in drug discovery for virtually all other therapeutic areas. In psychiatry, as discussed above, there have been virtually no examples of simple Mendelian inheritance and thus no single major genes have been discovered or exploited for developing a useful animal model of a common psychiatric disorder. Still, rare mutations causing neurodevelopmental disorders manifesting psychiatric symptoms (e.g. 22q deletion syndrome) for example have been used to study drugs that may prove useful for the more common and highly polygenic psychiatric disorders, but again with very limited if any success to date. The latter is in stark contrast to the more heritable neurological disorders where very useful and translatable animal models have been developed (e.g. for Huntington’s disease, amyotrophic lateral sclerosis [ALS], frontotemporal dementia [FTD] and Alzheimer’s Disease) (see below).

To our knowledge, few if any of the scores of molecular targets “validated” using these behavioral and genetic approaches and which have led to compounds reaching clinical development have been successfully developed for an actual psychiatric disorder. To make matters worse, in most cases there is limited data to actually confirm that the novel drug being studied actually resulted in the desired pharmacological action in the brain. In many cases it is not known whether a negative clinical study with such compounds provides evidence against the hypothesis being tested or simply reflects the fact that for reasons of poor dose selection or accessibility to the brain, the MOA in question was never evoked. Additionally, both increases and variability in placebo response rates have resulted in hugely expensive ultimately uninterpretable trials that did not meet their primary endpoints for studies of antidepressants in mood disorders and even in the case of antipsychotics for treating schizophrenia [8]. Moreover, in most studies the placebo response is 60-80% of the response observed with active drug! Not surprisingly, the failure to establish the clinical utility of literally dozens of compounds brought forward on the basis of such approaches has dampened enthusiasm by large pharmaceutical companies to make the substantial R&D investments required to discover and develop novel treatments for psychiatric disorders [9], despite the great medical and commercial success of the first and second-generation psychiatric drugs.

With respect to the economic incentives necessary for the substantial R&D investments required to discover new medicines, we can attest that these still exist for psychiatric drugs, especially if new and improved versions can be developed. In fact, the limited “effect sizes“ (poor overall efficacy) and troubling side effect profiles and tolerability of many, if not most, of the currently marketed psychiatric drugs make this a highly desirable business proposition, i.e. if it was not for the high attrition/failure rates described above. Thus, in our opinion, it is the comparatively low probability of technical success [9] coupled with, and in good part due to, our limited knowledge of disease biology that makes psychiatric drug development so risky and thus unappealing to the larger pharmaceutical companies, many of whom ironically were historically quite active and successful in the field of developing psychiatric drugs. This contrasts with many other therapeutic areas that currently compete for R&D investments, such as oncology, diabetes, cardiovascular and inflammatory diseases where we have a much better understanding of disease biology and many compelling drug targets. Today, with only a few exceptions, psychiatric drug discovery is now the purview of only a handful of smaller more risk-taking biotech companies and a few academic drug discovery programs. Hopefully, this picture will change as more compelling and less risky approaches to psychiatric drug R&D emerge.

## The solution

Given the substantial challenges outlined above, and notwithstanding the huge unmet medical need given the limitations of current psychiatric drugs, as well as the lessons learned from the last nearly three decades of rather dismal R&D productivity in psychiatry, we believe there are a number of opportunities to accelerate psychiatric drug discovery and development. We will outline and exemplify several of these opportunities below:

### Focus on disease biology

As emphasized above, our very limited understanding of either the etiology or pathophysiology of the major psychiatric disorders makes discovering highly effective drugs very challenging. Academic research on basic disease biology using all of the powerful tools available today (mentioned above) as well as the new tools on the horizon, some of which may emerge from the BRAIN initiative [10], must be adequately prioritized and funded. While progress has been slow in psychiatry, there are good examples of how a better understanding of disease biology (etiology and/or pathophysiology) can lead to successful drug discovery and development. Alzheimer’s disease (AD) is the most common form of dementia and is currently a uniformly fatal illness. Like many common and complex medical disorders there is now an impressive body of work that has unequivocally identified important genetic risk factors (e.g. apoE4 and TREM2) and even causal genes (e.g. APP, PS1) for subgroups of patients [11]. Importantly, large aligned investments delivered a variety of highly validated/qualified noninvasive biomarkers (e.g. PET/MRI neuroimaging, CSF and blood proteomics) [6] of the well described neuropathological hallmarks of the disease (amyloid plaques, neurofibrillary tangles, neuroinflammation and neurodegeneration) [12]. With these biomarkers to diagnose the disease at its earliest stages and follow progression from a preclinical or asymptomatic stage through a prodromal phase (MCI) to later stages, we can rationally discover and develop disease-modifying treatments for AD. The first disease-modifying drugs are just now being approved by the FDA and while far from perfect we submit that there are now at least a dozen drug targets for AD hotly being pursued by the biopharmaceutical industry that in our view will likely result in effective secondary and eventually primary disease prevention therapeutics [13]. This represents one of the best examples, including the kind of investments required, of how a better understanding of disease biology can enable drug discovery for a common and complex neuropsychiatric disorder. There are many other examples of inherited neurodegenerative disorders with simple Mendelian inheritance and where the identification of causal genes has or will undoubtedly lead to potential disease-modifying treatments. However, we fully acknowledge that such disorders are not typical of the more complex psychiatric syndromes and will still require novel and technically challenging approaches to correct (e.g. gene therapy). Nonetheless, as mentioned above, mutations causing even rare genetic neurodevelopmental disorders that have manifest psychiatric symptoms (e.g. psychosis) might provide valuable clues as to potential new drug targets for the more common disorders [14].

Another example of successful rational drug discovery in psychiatry is the recent discovery and development of brexanolone for treating postpartum depression (PPD) Brexanolone is a proprietary formulation of allopregnanolone, the major metabolite of progesterone and shown years ago to be a very potent positive allosteric modulator (PAM) of GABA_A_ receptors [15]. The increase in serum and brain progesterone and allopregnanolone levels that occurs during pregnancy and the sudden drop in allopregnanolone levels following childbirth have long been hypothesized to trigger PPD. An elegant series of preclinical experiments by Maguire and Mody [16, 17] provided support for this hypothesis by showing that the expression of GABA_A_ receptors (notably α and δ subunits) in critical brain regions is markedly reduced during pregnancy. Following parturition these receptors have to readjust to the normal very low levels of allopregnanolone observed in the absence of the pregnancy. Additional mouse experiments revealed a role for extra-synaptic GABA_A_ receptor δ subunits involved in maintaining excitatory: inhibitory (E/I) balance in the brain in maternal mice [17]. When the E/I balance is altered by genetically removing these δ subunits, abnormal maternal behaviors (poor nesting and nursing behavior and even infanticide) are observed following pregnancy and importantly these behaviors can be effectively treated with neurosteroids like allopregnanolone [16]. These findings led to a series of clinical trials in women with PPD where infusion of brexanolone over 60  hours followed by gradual tapering was shown to rapidly improve mood and anxiety symptoms in approximately 70% of women with PPD [18]. In many PPD patients the antidepressant effects of brexanolone were quite rapid and dramatic. And currently, given the issues attending prolonged IV administration, an oral synthetic neuroactive steroid zuranolone is being developed for both PPD and even MDD, with encouraging early clinical data [19]. In this example, clinical and preclinical evidence pointed to a targetable pathophysiology. We mention this because such a fundamentally empirical approach combined with the use of biomarkers to identify homogenous subgroups may prove a more successful way to uncover targetable pathologies than currently applied genetic strategies. The latter may hopefully one day constitute an additional source of targetable pathologies and importantly in potentially predicting drug response.

### Focus on sub-syndromal therapeutic targets

A focus on symptom domains rather than overall syndromal improvement in complex psychiatric diseases constitutes a major opportunity with recent adoption by both academia and industry. The most widely and arguably most misunderstood articulation of this general approach is captured in the Research Domain Criteria (RDoC) framework developed with NIMH funding [20]. Cognitive impairment associated with schizophrenia (CIAS) can be viewed as falling into this category: the domain of cognition in schizophrenia. While it is true that industry has funded many unsuccessful studies in CIAS we do not see this as an argument against focusing on this important and disabling symptom domain, since these earlier studies were undertaken in the absence of any strong evidence in humans of a quantitative brain effect or MOA that might reasonably be expected to lead to cognitive improvement. There have been and likely will be additional studies in schizophrenia and other psychiatric disorders which can be recast in the RDoC framework. As an example at least one industry sponsored study is pursuing “apathy” associated with Alzheimer’s disease [21]. It is anticipated that as the field matures and our ability to correlate objective brain measures (biomarkers of brain function) with clinical symptom domains, the probability of success of matching a specific molecular mechanism with a clinically-relevant therapeutic domain will increase.

If a reasonable case can be made that a drug effect on brain chemistry or function raises the probability of a beneficial therapeutic effect, then an expensive clinical study can be justified. One of the best examples of this to data in the field of psychiatry is provided by studies on a kappa opioid receptor (KOR) antagonist originally developed for depression by Eli Lilly. NIMH funded a study showing that this KOR antagonist can alter neural circuits involved in response to a reward task selected as the measure best suited to assess the domain of “hedonic response” [22]. Following positive findings using this biomarker, Johnson and Johnson, carried out a Phase 2 study with the KOR antagonist that had earlier been discontinued for treatment of depression. Positive results with atipracant as the compound is now called led to an current Phase 3 study that may lead to the first approved KOR antagonist for depression [23]. Nonetheless, by only advancing agents to Phase 2 trials which have been established to affect a brain process which can reasonably be linked to an important symptom domain, the success rates of such clinical trials will undoubtedly improve.

### Focus on experimental therapeutics (in homo veritas)

Despite the sophisticated science and tools the field of psychiatry now has at its disposal but considering all of the challenges highlighted above, the value of expeditiously obtaining actual clinical data in well characterized patient populations cannot be overstated. It is our view that there needs to be considerably more investments made to study mechanistically novel compounds in disorders where core symptoms can be reliably assessed along with the use of quantitative biomarkers. We refer to this broadly as “experimental therapeutics.” We would add that while seemingly heretical in psychiatry, even small uncontrolled studies can be informative. In fact, brexanolone was initially studied in a small open label study in women with post-partum depression (PPD) and the very encouraging results led quickly to more definitive placebo-controlled studies to confirm its efficacy and safety profile. In such open label studies, we feel that Type 2 errors (false negative results) are less likely to occur then Type 1 errors (false positive results) and therefore time and resources can be saved by conducting even small open label proof of concept (PoC) studies. If a given compound fails to show any activity in even a small open label study, it is unlikely to work in a larger placebo-controlled study. Moreover, the clinical observations gleaned from studies carried out in patients with psychiatric syndromes can also lead to additional more fruitful hypotheses The KOR antagonist anhedonia example cited above is a good case in point.

Another example of how astute clinical observations can lead to mechanistically novel approaches to treating psychiatric disorders is the case of xanomeline. Xanomeline is a muscarinic acetylcholine receptor agonist discovered in the early 1990’s and initially developed by Eli Lilly to improve cognition in patients with Alzheimer’s disease. In a large phase 2 study in AD patients Lilly investigators serendipitously observed that disruptive behavioral and psychotic symptoms (seen in approximately 30% of the patients) improved on xanomeline vs placebo [24]. While xanomeline modestly improved cognition, its effect in reducing psychotic symptoms was quite dramatic. It should be remembered that this antipsychotic activity was not shown by a pre-specified primary or secondary endpoint and thus would never pass rigorous statistical scrutiny. The results were nonetheless striking enough, like many serendipitous findings in psychiatry, to invest in a small, placebo-controlled study in schizophrenic patients with positive results [25] setting in motion the development of a completely novel drug (and MOA) to treat schizophrenia. The peripheral cholinergic AEs of xanomeline, especially the GI side effects, argued against further development of xanomeline. But considerable work in both academia and industry to better understand the exact MOA underlying xanomeline’s antipsychotic properties [26] provided the basis for developing a completely novel antipsychotic drug. These studies catalyzed parallel approaches to both improve the tolerability of xanomeline by co-administering it with a peripherally-restricted muscarinic receptor antagonist (trospium) as well as attempts to find more subtype-selective muscarinic receptor agonists devoid of the GI and other side effects of xanomeline. As a result, several recent placebo-controlled pivotal trials of xanomeline-trospium (KarXT) have confirmed the rather robust antipsychotic effects of xanomeline in patients with schizophrenia [27] and another recently completed phase 1 trial of emraclidine, a M4-selective muscarinic receptor allosteric agonist, suggests that it too may also have antipsychotic properties [28]. Importantly, these findings may eventually lead to one of the first non-D2 dopamine receptor blocking antipsychotic agents (among other compounds in Phase 3 studies) and devoid of the troubling side effects of the current standards of care such as weight gain, sedation, EPS, hyperprolactinemia and a risk of developing tardive dyskinesia [29]. Two other examples of this serendipitous approach to drug discovery in psychiatry immediately come to mind. The first is the observation that the dissociative anesthetic ketamine, later shown to be an NMDA receptor channel blocker, produced mood-elevating effects in some patients with schizophrenia as well as the more general finding of exacerbation of psychotic symptoms [30]. The mood-elevating properties of ketamine were unanticipated and subsequently led to ketamine’s use as an antidepressant in MDD/TRD and now in multiple other psychiatric disorders. This initial serendipitous finding has been exploited to come up with various ketamine-like drugs and in fact the S-isomer of ketamine (esketamine) has recently been approved by the FDA to treat treatment-resistant depression. The second example is related to the broader class of psychedelic drugs, including psilocybin and LSD. The antidepressant properties of these psychedelic drugs were initially and accidentally observed in essentially open label studies designed to relieve anxiety and depression in terminal cancer patients [31, 32]. These findings have led to multiple trials of both psilocybin and LSD in MDD and a large body of work on the underlying MOA of these drugs, with the hope of possibly finding non-psychedelic versions for more routine use [32]. While the issues surrounding the science and clinical utility of psychedelic drugs to treat various psychiatric disorders is well beyond the scope of this review their re-emergence for therapeutic purposes provides another example of the value of open label observational and subsequent placebo controlled clinical trials of novel experimental agents.

Taken together, with the examples of brexanalone and xanomeline, the experience with ketamine and psychedelic agents further underscores the value of studying compounds in humans and in the importance of astute clinical observations of symptom domains outside of a traditional primary outcome measure. While perhaps a less elegant approach to drug discovery than ideal (vide supra) we believe the field needs to embrace more experimental therapeutics and ideally by also incorporating the biomarker and sub-syndromal assessments described above. And there has been progress in terms of signal detection in psychiatric and other syndromal CNS trials captured in an ongoing series of position papers generated and listed by the International Society for CNS Clinical Trials and Methodology [33] which are already impacting clinical study design in ways that increase interpretability. Studying novel compounds in humans with psychiatric disorders already has and will, for all the above reasons, likely still lead to unanticipated findings and either directly or indirectly to mechanistically unique agents. Finally, we submit that establishing NIMH funded clinical research networks which should include clinical research training as has been done by the NCI will accelerate our ability to find and objectively characterize novel agents.

### Focus on biologically homogenous subgroups

There is now widespread acceptance that for common and complex psychiatric syndromes investigational drugs are unlikely to work well in all patients and identifying subgroups of responders and nonresponders will likely to be necessary to find better agents. Such stratification can be provided by a range of biomarkers based on measures of brain function. The most mature example of the utility of such biomarkers as a basis for patient selection for clinical trials is the aforementioned utilization of PET, serum, and cerebrospinal fluid biomarkers for measuring amyloid and tau pathology in Alzheimer’s disease which are now used routinely to diagnose patients and to monitor disease progression in clinical trials [12]. Projects to advance stratification biomarkers for a range of neurodegenerative disorders are underway through the Neuroscience Steering Committee of the Foundation of the National Institutes of Health Biomarkers Consortium [34]. The potential of similarly useful biomarkers in schizophrenia is the focus of the Schizophrenia Spectrum Biomarkers Consortium [35] which, based on recent genetic findings, seeks to test the hypothesis of abnormal complement function in schizophrenia as well as the Accelerating Medicines Partnership® Schizophrenia (AMP® SCZ) [36], an advocacy catalyzed public:private consortia effort aimed at characterizing multimodal biomarkers and clinical assessment strategies to better predict the trajectory of individuals at clinical high risk (CHR) for psychosis to enable studies of novel agents for early intervention in schizophrenia.

Another source of potential functional biomarkers may emerge from investments in differentiating patient-derived inducible pluripotent cells (iPSCs) into various types of neurons and even small “brain-like” organoids [37]. Many technical issues remain but multiple efforts are underway to see if these iPSC-derived neurons from individuals with defined psychiatric disorders can be used as in vitro test systems that allow one to predict whether or not that individual might respond to a specific drug such as lithium [38].

Functional biomarkers derived from EEG or fMRI measures are also showing promise for psychiatric disorders even in the absence of a known pathophysiological process. For instance, the B-SNIP consortium has found that differential EEG responses underpin three different biotypes of individuals who all present with psychotic symptoms and meet schizophrenia, schizoaffective or bipolar diagnoses [39]. A study is underway to see if such EEG informed subtyping predicts preferential response to clozapine [40] Similarly, analyses of EEG data generated by a large study of SSRI response in depression (the NIMH funded EMBARC) study using sophisticated machine learning and artificial intelligence methods revealed patterns that differentially predict either drug or placebo response [41]. And a consortium effort is focused on validating EEG methods as drug development tools [42].These and related AI approaches which utilize both EEG and clinical and task performance data form the basis of an approach taken by at least one company, Alto Neuroscience [43], to identify “responder” subgroups of patients to a variety of agents which have failed to separate from placebo in more traditional studies. Utilizing fMRI to measure brain activity and neural circuits in humans to bridge from optogenetic studies of circuit function in rodents has resulted in another set of predictors of antidepressant response [44]. Although it is too early to know which of these biomarkers or combination of biomarkers, if any, will prove most useful with regard to subgrouping of patients for clinical studies, early data suggests that one or more will prove successful. And, as noted above, a biomarker that proves useful in a clinical study may also point the way to a pathological process that can be targeted for novel drug development.

### Focus on combination treatments

It is also likely, given the polygenic and complex nature of psychiatric syndromes that drugs displaying considerable polypharmacology or combinations of drugs with distinct MOAs may work best for many, if not most, psychiatric disorders. The example of clozapine for treatment-resistant schizophrenia stands out as a serendipitous illustration of this possibility. It was only because there was a clinical impression of superiority in terms of efficacy to other antipsychotics that clozapine with all of its attendant side effects and risks was “rediscovered” and developed into the important therapeutic agent that it remains. At therapeutic doses, clozapine has been shown to affect not only various dopamine receptors but also several subtypes of serotonergic and noradrenergic receptors along with a myriad of other potentially relevant activities [45]. And, in another example of possible benefits of engaging more than one mechanism, earlier depressed inpatient studies showed superior efficacy of dual action TCAs over SSRIs [46].

The strategy of testing novel agents in combination with an existing standard treatment in patients who have shown at best a partial response is now being widely pursued. Here both clinical science and business opportunities align. This approach incorporates both the possibility that an added drug would treat a specific domain as in the CIAS example mentioned above as well as the possibility that overall global efficacy. As we learn more about potential additive and synergistic effects of combining more than one MOA in various preclinical models and systems and apply the tools of quantitative systems pharmacology [47] testable hypotheses on the benefits of combined treatments are emerging. As part of this hypothesis generation, biomarker-identified subgroups of patients may well help identify where a particular combination might be most effective. As noted above, biomarker development is promising in this regard and therefore considerably more than just “shot gun” empiricism in deciding what combinations to explore and in what patients. It should be noted that drug combination strategies are now the norm for treating most cancers, something we believe will become common place in psychiatry as mechanistically unique psychiatric drugs emerge. We would advocate considerably larger investments in biomarker discovery and qualification by NIMH/NIH, which has already recognized some of these opportunities and expanded investments in public private partnerships [48, 49], as the private sector is unlikely to make such investments at the scale required.

### Hope for black swan events

We have focused our attention on more or less traditional small molecule drug discovery approaches but of course the brain is an electrochemical organ and altering brain chemistry via small molecule drugs is but one way to potentially treat serious psychiatric disorders. We should remain opportunistic and open to the possibility that better unanticipated approaches may emerge. A similar “black swan“ event has occurred in oncology just over the past decade where very targeted approaches to treating cancer based on well accepted somatic driver mutations (e.g. gain of function receptor tyrosine kinase mutations) were dominant but in many cases have now been superseded by the advent of immuno-oncology (IO) which has been shown to be very effective for certain cancers where boosting immune cell function can dramatically inhibit tumor growth and increase survival [50]. These IO drugs have revolutionized the treatment of certain cancers (e.g. melanoma). Are there similar potential “black swan events“ awaiting psychiatric therapeutics? We will mention only two possibilities here. The first is the potential of developing large molecule therapeutics, such as antibodies, for treating psychiatric disorders. New antibody technology, utilizing for example receptor-mediated trancytosis, has recently been developed which allows larger proteins like antibodies normally unable to readily cross the blood:brain barrier to any appreciable extent, to more efficiently access the brain [51]. This technology is being adopted for neurodegenerative disorders primarily but could open up the field of antibody therapeutics to psychiatry as well. Finally, advances in “precision“ deep brain stimulation (DBS) while too invasive for routine use for most psychiatric disorders [52] suggest that the development of noninvasive brain stimulation techniques (eg deep TMS-like technology) could potentially revolutionize psychiatric therapeutics and preliminary data in mood disorders are encouraging [53].

## Summary

While progress in discovering and successfully developing truly novel (new and improved) psychiatric drugs has been slow and disappointing, in large part due to our rather limited understanding of the etiology and pathophysiology of the major psychiatric syndromes, we remain optimistic and believe there is now “light at the end of the tunnel.” Several novel, mechanistically speaking, drugs for depression and schizophrenia are on the horizon and there is renewed interest on the part of both large and small biopharmaceutical companies to invest the necessary resources, intellectual and financial, required. While the field of psychiatry works to better define the etiology and pathophysiology of the major psychiatric disorders there is still much to be done in the absence of such information and we have highlighted several areas of fruitful pursuit in this review. Given the complexity of these brain disorders we must reduce their unquestionable heterogeneity in order to discover precision medicines that will be more effective, not just for reducing core symptoms, but in improving functional outcomes for the many people who are still disabled with current treatments and especially for those who will unfortunately otherwise succumb to their illness.
